# Comparison of pneumococcal colonization density among healthy children and children with respiratory symptoms using real time PCR (RT-PCR)

**DOI:** 10.1186/s12866-022-02442-z

**Published:** 2022-01-20

**Authors:** G Vidanapathirana, A L S K Angulmaduwa, T S Munasinghe, E W M A Ekanayake, P Harasgama, S T Kudagammana, B N Dissanayake, L V C Liyanapathirana

**Affiliations:** 1 Faculty of Medicine, University of Peradeniya, Kandy, Sri Lanka; 2 Department of Microbiology, Faculty of Medicine, University of Peradeniya, Kandy, Sri Lanka; 3 Department of Paediatrics, Faculty of Medicine, University of Peradeniya, Kandy, Sri Lanka

**Keywords:** Children, Colonization, Culture, Pneumococci, RT-PCR

## Abstract

**Background:**

Nasopharyngeal colonization is considered a necessary step in the initiation of pneumococcal diseases. Real time PCR (RT-PCR) is an alternative approach for the identification and quantification of pneumococci directly from samples.

**Objectives:**

To compare pneumococcal detection rates using culture-based method versus RT-PCR direct detection and to quantify pneumococcal colonization in two study cohorts (healthy children and hospitalized children with respiratory symptoms) using quantitation through RT-PCR.

**Methodology:**

A total of 101 nasopharyngeal swabs (NPS) from healthy children and 183 NPSs from hospitalized children with respiratory symptoms were included in the study. None of the children were vaccinated. All children were between 2 months to 2 years. In parallel to routine culture and identification, a RT-PCR assay targeting the lytA gene was done.

**Results:**

Considering all 284 samples tested, colonization rate by conventional culture was 41.2% (*n* = 117) while positive colonization using RT-PCR was 43.7% (*n* = 124). The colonization rate detected by RT-PCR in the healthy cohort was 33.7% (*n* = 34) and it was 49.2% (*n* = 90) in the hospitalized cohort. It was 37.6% (*n* = 38) and 43.2% (*n* = 79) for the two cohorts by culture. The mean Cq value for the healthy cohort is 29.61 (SD 2.85) and 28.93 (SD 3.62) for the hospitalized cohort.

With the standard curve obtained from amplifying a dilution series of control DNA, the mean amount of genomic DNA copy numbers detected in children with respiratory symptoms was log10 7.49 (SD 1.07) while it was log10 7.30 (SD 0.23) in healthy children and the difference was not statistically significant.

**Conclusions:**

The overall colonization rate was higher when detected using RT-PCR compared to culture. However, it was lower in the healthy group when detected with RT-PCR compared to culture. Even though there was a higher detection of pneumococcal colonization density in children with respiratory symptoms, this was not significantly higher unlike many previous studies. Therefore, the use of RT-PCR to detect pneumococcal colonization needs further evaluation with careful analysis of interpretation and confounders.

## Introduction

*Streptococcus pneumoniae* or pneumococcus is a pathogenic bacterium which can cause a variety of diseases ranging from non-invasive diseases such as otitis media, sinusitis to invasive diseases including pneumonia, meningitis, and sepsis. According to the World Health Organization (WHO), pneumococcal diseases were responsible for an estimated 317,300 pediatric deaths in 2015 [1]. Nasopharyngeal colonization of pneumococci has been identified as a prerequisite for the initiation pneumococcal disease and transmission of pneumococci [2]. Pneumococcal colonization rates differ globally according with socioeconomic factors and age [3]. It is highest among children below the age of 5 years [4]. In this age group too, it is found to vary between countries [4–8].

Laboratory identification and typing of pneumococci is performed using conventional methods based on phenotypic characteristics of pneumococci, serological tests and the molecular based techniques. The conventional method of identifying *Streptococcus pneumoniae* is through culture and detection of optochin sensitivity and bile solubility in presumptively identified isolates [9]. However, attributing aetiology is difficult, particularly when isolates are from nonsterile sites, where pneumococci may be found as part of the normal flora. This is confounded by identifying isolates that express different phenotypic characteristics. According to the published data, Deoxycholate solubility (Bile solubility test) has been associated with a sensitivity of 98% and a specificity of 100%. However, pneumococcal isolates that are bile insoluble have also been reported [10].

Identification of pneumococcal aetiology of infections can be difficult since pneumococci are found as normal flora in children. Several studies have reported that high pneumococcal density in the nasopharynx is associated with pneumonia in children [11, 12] in addition; colonization with multiple pneumococcal serotypes is common in children in low- and middle-income countries [13].

There are only a few published data on quantitative analysis of pneumococcal colonizing density in children. A multicenter study conducted in Bangladesh, the Gambia, Kenya, Mali, South Africa, Thailand, and Zambia to find the density of upper respiratory colonization with *Streptococcus pneumoniae* among children less than 5 years, hospitalized with severe pneumonia revealed an association between pneumococcal colonization density and microbiologically confirmed pneumococcal pneumonia [14]. There are other similar studies which focused on the pneumococcal colonizing density and the prevalence of upper respiratory tract illnesses in children [12, 15, 16]. In addition to that, some studies have compared the pneumococcal densities in children before and after the implementation of pneumococcal conjugate vaccine [17, 18]. However, many of these studies have been done in well controlled matched groups than in real world, clinical settings.

The main aim of the present study was to evaluate the pneumococcal colonizing density in healthy children and hospitalized children with respiratory symptoms using RT-PCR, in a vaccine naïve population. Further, comparison of pneumococcal detection rates using culture-based method versus RT-PCR direct detection was also done. We also aimed to see if serotyping can be done directly from extracted samples as this gives the additional advantage of identifying co-colonization, with the comparatively less expensive conventional PCR.

## Methodology

For this study, we used a sub group of participants of a larger study. For the samples used for this analysis both culture based and RT-PCR based methods were used to identify pneumococcal colonization [19]. We selected samples taken from 101 healthy subjects between 2 months and 2 years (24 months) of age attending immunization clinics and 183 children being hospitalized with respiratory symptoms (cough, runny nose, difficulty in breathing, with or without fever as documented in the medical chart) at Teaching Hospital Peradeniya, Sri Lanka from October 2017–November 2018 for this sub-analysis. Samples were selected from the original 900 samples randomly [19]. These nasopharyngeal swabs were collected using an age-appropriate nylon tipped flocked swab (Hydraflock_, Hardy Diagnostics, U.S.A). NPS was immediately placed in a sterile screw-capped tube with STGG medium, transported to the laboratory and stored at -80 °C till testing. Samples from the hospitalized group were collected within 48 h of hospitalization.

Participants were recruited after obtaining informed written consent from a guardian. Ethical approval for this study has been obtained from the Ethics Review Committee of Faculty of Medicine University of Peradeniya, Sri Lanka (2016/EC/63). None of the study participants were vaccinated.

### Quantification of pneumococci

An aliquot of randomly selected samples (*n* = 101 from the healthy carriage group and *n* = 183 from the samples collected from hospitalized children), were used for this component of the study. QIAamp® DNA mini kit (Qiagen) was used to extract DNA from the STGG aliquoted. Thawed samples were mixed with 0.1% sodium deoxycholate incubated for 1 h at 37 °C before following manufacturer’s instructions for the rest of the procedure [20].

Real time PCR was performed as previously described by Carvalho et al. [21]. Protocol was followed as given in the CDC Streptococcus lab protocol section using Promega GoTaq® Probe RT-PCR master mix [22].

As positive control, genomic DNA extracted from *Streptococcus pneumoniae* ATCC 49,619 was used. This DNA was quantified using Nanodrop and a tenfold dilution series was prepared. Each dilution was run in duplicate. Cq value of the control dilution series was used for identification of PCR efficiency as well as to quantify the DNA concentration [23].

### Semi quantitative culture method

All samples were thawed, vortexed, and processed microbiologically as recommended by the WHO pneumococcal carriage working group [24]. Samples were plated on quality-controlled sheep blood agar (SBA) and incubated at 37 °C in 5% carbon dioxide. Plates were incubated for 48 h before discarding. Alpha hemolytic colonies were purified and pneumococci were identified by optochin sensitivity (incubated at 5% CO2) and bile solubility testing. Confirmed pneumococcal isolates were stored in STGG at -80 °C.

Semi-quantitative colony count was evaluated according to the growth of pneumococci along the streak lines.

**Fig. 1:**
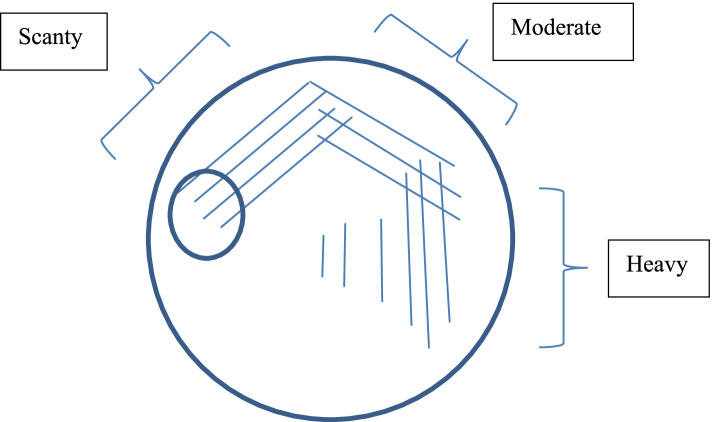
Semi-quantitative colony count

Growth of alpha haemolytic colonies on the SBA plate was quantified as scanty, moderate and heavy according to the scheme given in Fig. 1.

### Direct detection of pneumococcal serotypes

An attempt was made to detect co-colonization of different serotypes using the samples that tested positive for the *lyt*A real time PCR. Direct Serotyping of the pneumococci in nasopharyngeal samples was performed using the sequential multiplex PCR protocol recommended by the CDC, Altanta [25]. However, only conventional multiplex-PCR was performed. As the multiplex PCRs failed to yield the *cps* band in many of these samples, a single conventional PCR for *cps* locus was done for those samples. In addition, single PCR was run for serogroup 6 and serotype 19F in the samples that tested positive for *cps* by conventional PCR.

## Results

### Pneumococcal colonization rates and densities

A total of 284 nasopharyngeal swabs were tested with RT-PCR. 101 of the samples were from healthy children while 183 were from hospitalized children. RT-PCR positivity as defined by a Cq value of =  < 35 was 43.7% (*n* = 124) overall, while it was 33.7% (*n* = 34) among healthy children while it was 49.2% (*n* = 90) among the hospitalized children (*p* = 0.013, Chi-square test). Among these 284 participants, 117 (41.19%) were detected to be colonized with pneumococci using culture-based method. The colonization rate by culture among healthy children was 37.6% (*n* = 38) while it was 43.2% (*n* = 79) among the hospitalized children (*p* = 0.217, Chi-square test).

Considering the RT-PCR positive samples, the mean Cq among healthy children was 29.61 (SD 2.85) while the mean Cq among the hospitalized children was 28.93 (SD 3.62). This difference was not statistically significant (*p* = 0.328, independent sample t test).

With the standard curve obtained from amplifying a dilution series of control DNA [21] the mean amount of genomic DNA copy numbers detected in children with respiratory symptoms was log_10_ 7.49 (SD 1.07) while it was log_10_ 7.30 (SD 0.23) in healthy children (Table 1). The difference was not statistically significant (*p* = 0.354, independent sample t test).

**Table 1:** Mean Cq values and the genomic copy numbers of the two study cohorts

**Study cohort**	**Mean Cq value**	**Mean Genomic DNA copy number**	**Difference**
Cohort A (Healthy babies from the immunization clinic) (*n* = 101)	29.61 ± 2.85	Log10 7.30 ± 0.23	*p* = 0.354, independent sample t test
Cohort B (Hospitalized babies cohort) (*n* = 183)	28.93 ± 3.62	Log10 7.49 ± 1.07	

### Colonization by RT-PCR vs semi-quantitation on culture

There were 21 participants with RT-PCR positivity where culture did not reveal a *Streptococcus pneumoniae*. There were also 14 participants where culture was positive for *Streptococcus pneumoniae* and RT-PCR was negative (Table 2). These 14 patients had yielded serotype 19F (*n* = 4), 6A (*n* = 2), 6B (*n* = 1), 11A/D (*n* = 1), 9 N/L (*n* = 1), *cps* and *lytA* negative isolates (*n* = 3) and *cps* negative but *Lyt*A positive isolates (*n* = 2) by culture.

**Table 2:** Comparison of semi quantitative culture and RT-PCR results

Culture result	RT-PCR positive (Cq = < 35) (*n* = 124)	RT-PCR negative (Cq > 35) (n-160)
No *S. pneumoniae* isolated (*n* = 167)	21 (12.6%)	146 (87.4%)
Heavy growth of *S. pneumoniae* isolated (*n* = 31)	31 (100%)	0
Moderate growth of *S. pneumoniae* isolated (*n* = 27)	25 (92.6%)	2 (7.4%)
Scanty growth of *S. pneumoniae* isolated (*n* = 59)	47 (79.7%)	12 (20.3%)

^*^Percentages given with the culture result as the denominator

### Identification of serotypes/serogroups and co-colonization

Of the 124 *lytA* RT-PCR positive samples, only 85 (68.5%) were positive for the *cps* gene by conventional PCR targeting *cps* gene only. When the RT-PCR positive samples were tested with CDC1 multiplex PCR, only 54 (43.5% of the samples yielded a cps band while 83 (66.9%) samples yielded a cps band in the CDC 2 multiplex PCR. Moving only to serotyping results the following serotypes/groups were identified by direct PCR using CDC1 and CDC2 multiplexes (Table 3).

**Table 3:** Identification of the serotypes

Serotypes/groups	Frequency	Percentage
Serogroup 6	18	14.5%
Serotype 19F	7	5.6%
Serotype 14	5	4%
Serotype 9A/V	1	0.7%
Serotype 23F	1	0.7%

Co-colonization was found in 2 samples (serotype 23F – serogroup 6 and serotype 19F – serotype 14). We performed single PCR for serotype 19F and serogroup 6 on the samples that had positive cps signals in the single PCR. In that, 23 samples were found to be positive for serogroup 6 while 17 samples were positive for serotype 19F. Four samples were found to contain both serogroup 6 and serotype 19F. Due to limited amount of sample, further testing was not attempted.

## Discussion

An overall colonization rate of 43.7% (124/284) was identified using the RT-PCR method among two groups of children aged between 2 months and 2 years in the present study. Healthy children had a colonization rate of 33.7% while children hospitalized with respiratory symptoms with or without fever had a colonization rate of 49.2%. Colonization rate as detected by culture among the participants was 41.19%. The higher colonization rate of the current sub-analysis is due to the higher sensitivity of the RT-PCR method.

There are several other studies which have reported pneumococcal colonization using RT-PCR method. Courtney, Piralam. et al. [16] and Wyllie AL. et al. [26] have identified 82% and 69% pneumococcal colonization rates in healthy baby cohorts. In addition to that, Barameht P. et al. [27] and Niclas J. et al. [28] have identified 57.2% and 62% pneumococcal colonization rates among hospitalized children. This difference might be due to the time lapse, differences in socio-demographic backgrounds of participants, geographical differences, the specimens used in the studies or the age difference in the study groups. Further, we have used a cut off for considering RT-PCR positivity; this may have led some of the high Cq value positives with lower colonization density to be considered as not colonized with pneumococci.

A higher pneumococcal colonization density was observed in the hospitalized children cohort. Some previous studies have detected a higher pneumococcal colonization density in hospitalized children with respiratory symptoms and pneumococcal diseases [12, 15, 29, 30] while others have not [16].

Another study has evaluated the association of PCV immunization on the prevalence density of nasopharyngeal colonization by common, potentially pathogenic bacteria including *S.pneumoniae* [17]. They have observed a reduction in PCV7-serotype colonization which in turn had an impact on colonization prevalence and density of other bacterial species of the nasopharynx. However, none of our study population was vaccinated. As we did not have data on the final diagnosis of the patients or antibiotic use, we are unable to analyze the colonization density across disease categories or the effect of antibiotics on this result.

When comparing the conventional culture method and the RT-PCR method, *Lyt*A PCR was positivity rate was higher than culture positivity rate for *Streptococcus pneumoniae* in the hospitalized children cohort. This is expected given the sensitivity of the real time PCR assay [31]. However, there was a lower pneumococcal detection rate in the healthy baby cohort using the RT-PCR method than the conventional culture method. This might be attributed to the presence of lower colonization density leading to a lower concertation of DNA and the use of a Cq of 35 to define positivity. This highlights the need to carefully evaluate the interpretation used in detection of colonization with RT-PCR.

In addition to that, RT-PCR positivity correlated with semi quantitative culture, as demonstrated by others earlier [30].

A similar study has examined pneumococcal carriage dynamics, including density and multiple serotype carriage, in Indonesian infants during the first year of life [32]. The study has revealed an overall pneumococcal colonization rate of 22% by *lyt*A RT-PCR. In addition to that, 6B, 19F, 23F, 34, and 15B/C were identified as the common serotypes using the microarray technique. The mean pneumococcal colonizing density of the study cohort was 6.04 log10 GE/ml which is slightly lesser than to the colonizing density of the present study. However, multiple serotype carriage was observed in 98 samples which are relatively higher than to our study. Further, the study has revealed that multiple serotype carriage was associated with the higher pneumococcal density. We attempted to identify the pneumococcal serotypes using conventional PCR, on the extracts that yielded pneumococci through RT-PCR and the attempt failed, due to the differences in sensitivities among the two methods. We could not perform RT-PCR based direct serotyping, therefore, it is difficult to comment conclusively on the co-colonization rates. However, we could state that any study that aims to detect co-colonization with molecular methods should use RT-PCR or another high sensitivity method such as microarray instead of conventional PCR This area needs further diagnostic evaluation in real-world setting due to the costs associated.

There are some limitations of the present study. Although, an attempt was made to detect co-colonization of different serotypes using the samples that were positive with *lyt*A real time PCR, only conventional multiplex PCR was used due to limited funding. As the multiplex PCRs failed to yield the *cps* band in many of these samples, a single conventional PCR for *cps* locus was done for those samples. However, the positivity rate for this was also lower than for the real time PCR. Attempt to serotype directly using conventional multiplex PCRs gave unfavourable results. Use of single PCRs targeting the commonest serotypes identified by culture also gave rise to a relatively low positivity rate than expected based on culture isolate results. This is possibly due to the comparatively lower sensitivity rate of conventional PCR than real time PCR.

Although we have used *lyt*A RT- PCR to identify pneumococci, certain other bacteria such as oral streptococci can harbor the *S. pneumoniae lyt*A gene due to horizontal gene transfer [33]. Hence, a PCR technique with Spn9802- and Spn9828-specific primers, in combination with *lytA*- and *ply*-specific primers, has been recommended for identifying *S. pneumonia* [34].

Further, we did not have data on the clinical diagnosis of the patients with respiratory symptoms, therefore, a sub-analysis within this group could not be performed to identify any difference between the colonization densities in different disease groups. Further, we did not analyze the effect of antibiotic treatment on colonization density.

## Conclusion

In conclusion, present study revealed a higher pneumococcal colonization rate using the RT-PCR method comparing with the conventional culture method. In addition to that, a higher pneumococcal colonization density was reported in hospitalized children with respiratory symptoms which is in congruent with the previous studies, however the difference was not statistically significant unlike in some previous studies. Therefore, the use of quantitative RT-PCR along as a part of diagnostic algorhythms for aetiological diagnosis of pneumococcal diseases in children should be further evaluated. Further, we identified that the use of conventional PCR for direct serotyping on sample identified to contain pneumococci through RT-PCR, aiming to identify co-colonization is not an effective method.

## Data Availability

Data are available from the corresponding author on reasonable request.

## Abbreviations

RT-PCR: Real Time Polymerase Chain Reaction
NPS: Nasopharyngeal swabs
WHO: World Health Organization
STGG: Skim milk, Tryptone, Glucose, and Glycerin
CDC: Centre for Disease Control and prevention
SBA: Sheep Blood Agar
ARI: Acute Respiratory Illnesses
